# *Pseudomonas* cultivated from *Andropogon gerardii* rhizosphere show functional potential for promoting plant host growth and drought resilience

**DOI:** 10.1186/s12864-022-09019-0

**Published:** 2022-11-30

**Authors:** Soumyadev Sarkar, Abigail Kamke, Kaitlyn Ward, Eli Hartung, Qinghong Ran, Brandi Feehan, Matthew Galliart, Ari Jumpponen, Loretta Johnson, Sonny T.M. Lee

**Affiliations:** 1 Division of Biology, Kansas State University, Manhattan, KS USA; 2 Department of Biological Sciences, Fort Hays State University, Hays, KS USA

**Keywords:** *Pseudomonas*, Cultivation, Drought, Rhizobiome, Stress, Nitrogen

## Abstract

**Background:**

Climate change will result in more frequent droughts that can impact soil-inhabiting microbiomes (rhizobiomes) in the agriculturally vital North American perennial grasslands. Rhizobiomes have contributed to enhancing drought resilience and stress resistance properties in plant hosts. In the predicted events of more future droughts, how the changing rhizobiome under environmental stress can impact the plant host resilience needs to be deciphered. There is also an urgent need to identify and recover candidate microorganisms along with their functions, involved in enhancing plant resilience, enabling the successful development of synthetic communities.

**Results:**

In this study, we used the combination of cultivation and high-resolution genomic sequencing of bacterial communities recovered from the rhizosphere of a tallgrass prairie foundation grass, *Andropogon gerardii*. We cultivated the plant host-associated microbes under artificial drought-induced conditions and identified the microbe(s) that might play a significant role in the rhizobiome of *Andropogon gerardii* under drought conditions. Phylogenetic analysis of the non-redundant metagenome-assembled genomes (MAGs) identified a bacterial genome of interest – MAG-*Pseudomonas*. Further metabolic pathway and pangenome analyses recovered genes and pathways related to stress responses including ACC deaminase; nitrogen transformation including assimilatory nitrate reductase in MAG-*Pseudomonas,* which might be associated with enhanced drought tolerance and growth for *Andropogon gerardii.*

**Conclusions:**

Our data indicated that the metagenome-assembled MAG*-Pseudomonas* has the functional potential to contribute to the plant host’s growth during stressful conditions. Our study also suggested the nitrogen transformation potential of *MAG-Pseudomonas* that could impact *Andropogon gerardii* growth in a positive way. The cultivation of MAG-*Pseudomonas* sets the foundation to construct a successful synthetic community for *Andropogon gerardii*. To conclude, stress resilience mediated through genes ACC deaminase, nitrogen transformation potential through assimilatory nitrate reductase in MAG-*Pseudomonas* could place this microorganism as an important candidate of the rhizobiome aiding the plant host resilience under environmental stress. This study, therefore, provided insights into the MAG-*Pseudomonas* and its potential to optimize plant productivity under ever-changing climatic patterns, especially in frequent drought conditions.

**Supplementary Information:**

The online version contains supplementary material available at 10.1186/s12864-022-09019-0.

## Background

Global climate change is a serious concern, resulting in soil degradation, soil erosion, and impacts on soil health [1]. Climate change has severe impacts worldwide including in the USA, resulting in more frequent and prolonged droughts [2], gradually degrading the plant diversity and ecosystem functions [3]. The rhizobiome, microbial communities that are intimately associated with the plant rhizosphere [4, 5], is one of the key factors in maintaining ecosystem function, soil quality and plant health [6, 7]. The plant rhizosphere is one of the sites for plant–microbe and microbe-microbe interactions, governed primarily by root exudates [8]. Microbes in the rhizobiome can facilitate plant host nutrient and water uptake, element cycling (carbon, nitrogen, phosphorus), and other processes such as inducing plant growth, and enhancing plant drought tolerance that is beneficial to plants [9–11].

Rhizobiomes are instrumental in enhancing plant hosts’ resistance and resilience against abiotic stresses such as drought, salinity, and heavy metal exposure [12]. Therefore, with the more frequent and more extreme droughts events predicted in the global climate change scenarios in the future, it is essential to provide new insights into the mechanisms of how the rhizobiome may promote plant host resilience and response to stress. Reports show that bacteria populations can modulate the associated plant stress responses to environmental stresses [13, 14]. Plants respond to the above-mentioned stressors by modulating the level of various hormones, such as ethylene, which in turn induce the expression of stress-related proteins [15, 16]. However, when ethylene is produced more than its threshold level, it becomes unfavorable in terms of root/shoot proliferation and other growth parameters, hindering plant growth and development [17]. Bacteria-mediated 1-aminocyclopropane-1-carboxylate deaminase (ACCD) is able to mediate the enhanced resistance to biotic stressors and increased tolerance to abiotic stresses in their associated plant hosts [18–24], by breaking down ACC, an immediate precursor of ethylene resulting in plants resuming root/shoot growth [25, 26]. Although there are various studies that have dissected how climate change impacts the rhizobiome [13, 27–29], more concerted efforts are needed to provide insights into the mechanisms of how the rhizobiome can enhance the plant host resilience during drought-induced stress.

Previous studies have reported a clear contribution from plant-associated microbial members to plant growth and resilience during drought conditions [30–32]. Plant growth-promoting bacteria (PGPB) reportedly enhance plant growth during drought [33, 34], an observation attributed to the microbial nitrogen cycling and transformation in soil [35]. Therefore, candidate microbes capable of nitrogen transformation and increasing nitrogen availability in the rhizosphere have been the key targets in a growing number of experimental and observational studies that focus on the assembly of plant health promoting Synthetic Communities (SynCom) [36, 37]. SynComs have been successfully deployed to alter the plant phenotype, to enhance plant disease resistance and productivity [38, 39]. However, it is challenging and tedious to select optimal members of SynComs because of the lack of knowledge of the microorganisms that could impart favorable functions under stressful conditions [40]. Therefore, in identifying candidate microbes for SynComs, it may be more expedient to identify specific microbial functions and mechanisms rather than to depend solely on taxonomy.

Our long-term, ongoing research on the microbiome of dominant Great Plains prairie grass *Andropogon gerardii* (Big Bluestem) provided an excellent opportunity to acquire deeper insights into the microbial functional potential under abiotic stress [41–43]. There are three *A. gerardii* ecotypes (dry, mesic, and wet) that originated in Hays, Kansas ( averaged annual precipitation ~ 500 mm), Manhattan, Kansas (averaged annual precipitation ~ 870 mm) and Carbondale, Illinois (averaged annual precipitation ~ 1,200 mm), respectively [41, 42]. In this study, we attempted to elucidate the rhizosphere microbial functional potential from *A. gerardii* dry and wet ecotypes growing in Colby, Kansas, where the low precipitation defines a margin of the environment suitable for *A. gerardii* survival and growth. In Colby, the averaged annual precipitation regime is comparable to Hays, Kansas. We aimed to identify the *A. gerardii* rhizobiome associated microbial population(s) that are drought resistant or resilient, and to acquire insights into the microbial functions by: (1) recovering and cultivating microbes that existed in the ecotypes using media that promote drought-induced stress [41]; (2) obtaining genomic insights into drought resilient bacterial populations that can contribute to the nitrogen transformation. In this study, we combined cultivation and high-resolution genomic sequencing to identify microbial populations and their functional potential to enhance *A. gerardii* resistance and resilience during drought stress. The ultimate goal of this study is to identify bacteria populations and their functional potentials in synthetic communities (SynComs). In the previous study, we recovered gDNA directly from the soil to identify the bacterial and fungal populations under abiotic environmental stress [44]. The current study takes one step further to undertake a strategy to potentially construct operative SynComs in the future, by cultivating and selecting for a few potential candidate microorganisms that could be engineered for the benefit of the host.

## Results and discussion

### MAGs analysis, phylogenetic analysis, and identification of MAG-Pseudomonas

**Fig. 1:**
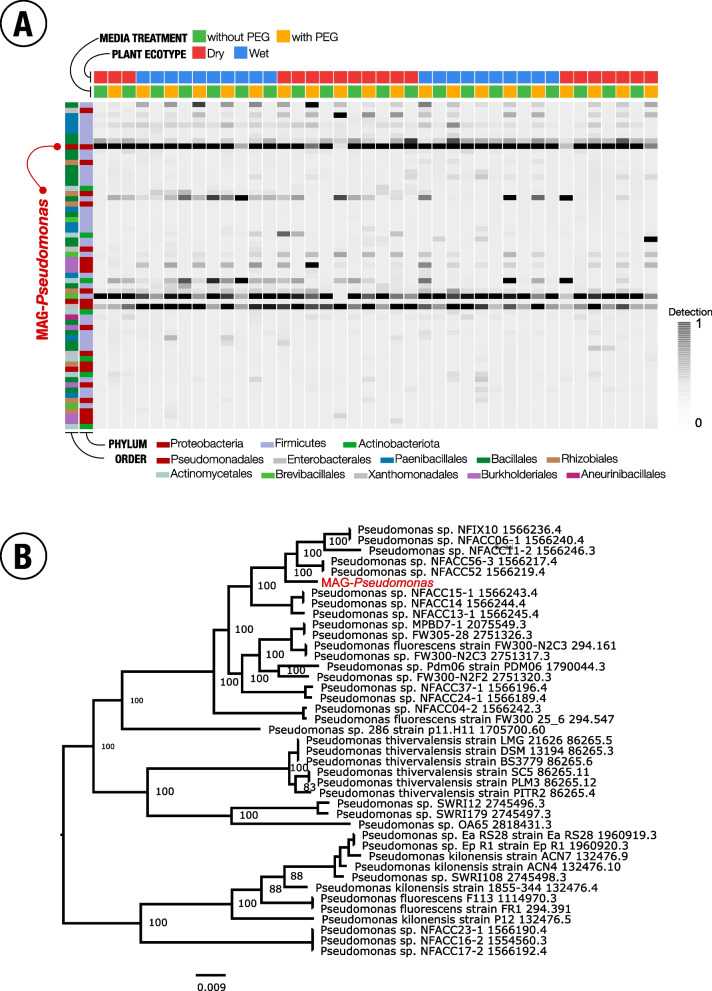
**A** Detection, showing reads recruited to the contigs of non-redundant metagenome-assemble genomes (MAGs) in the rhizosphere of dry and wet *Andropogon gerardii* ecotypes when cultivated in normal precipitation (without PEG) and under drought-induced conditions (with PEG). The darker the highlight represents higher detection in the samples*.* MAG-*Pseudomonas* was highly detected in all growing media conditions of both dry and wet ecotypic rhizosphere samples. **B** Phylogenetic analysis of MAG-*Pseudomonas* based on the 40 closely related whole genomes with small set of potential outgroup genomes. Tree scale indicated the length of the branches of the tree in terms of evolution. Note that *Pseudomonas fluorescens* F113 and *Pseudomonas fluorescens* FR1 are now known to be *Pseudomonas ogarae* sp. nov., nom rev., type strain F113^T^ (= DSM 112162^ T^ = CECT 30235.^T^). *Pseudomonas* sp. NFACC52 was the most closely related genome to MAG-*Pseudomonas*. The outgroup in the phylogenetic tree is *Pseudomonas* sp. NFACC23-1, *Pseudomonas* sp. NFACC16-2, and *Pseudomonas* sp. NFACC17-2

We cultured the *A. gerardii* rhizosphere microbial populations using the following samples and media—dry ecotype in R2A, dry ecotype in R2A with PEG, wet ecotype in R2A, and wet ecotype in R2A with PEG. We expected that the PEG-amended media would yield bacterial populations enriched with drought-resistant gene functions. We recovered an average of 173,480 ± 22,383 contigs, with N50 of 33,485 ± 2,526. The resolved metagenome-assembled genomes (MAGs) were 4.1 ± 1.3 Mbp had completion values of 89.4% ± 2.0%, and ~ 92.8% were annotated to the genus level (Supplementary Table S1). We recovered a total of 125 MAGs and generated a total of 63 non-redundant MAGs from the four conditions (Fig. 1A, Supplementary Table S1). MAGs that share > 95% average nucleotide identity were considered to be redundant MAGs. We identified 62 redundant MAGs, and those were excluded from downstream analyses. The dominant phyla among the non-redundant MAGs were Proteobacteria (*n* = 20), Firmicutes (*n* = 36) and Actinobacteria (*n* = 7).

Among the 63 non-redundant MAGs that we resolved, one of the clusters (consisting of four MAGs having > 95% ANI identity) was assigned to the genus *Pseudomonas*. We obtained 40 closest related *Pseudomonas* genomes using the Similar Genome Finder service, and observed that the MAG was phylogenetically close to *Pseudomonas sp.* NFACC52 (Fig. 1B, Supplementary Table S1)*.* The MAG-*Pseudomonas* was found to belong to the *Pseudomonas corrugata* subgroup within the *Pseudomonas fluorescence* species complex [45, 46]. We observed that the representative MAG (MAG_001; hereafter referred to as MAG-*Pseudomonas*) for this cluster was highly detected in all the culture conditions, and with their ubiquitous presence in the soil irrespective of the ecotype and drought stress, we hypothesized that MAG-*Pseudomonas* might be an important contributor in the rhizobiome associated with *A. gerardii*. Not commonly found, but rarely *Pseudomonas* has been identified in the *A.gerardii* rhizosphere [47]. *Pseudomonas spp.* are common in the rhizosphere of other plants, and reported to have important functions in modulating host performance [48–50]. MAG-*Pseusomonas* was found to be relatively close to the *Pseudomonas thivervalensis*. The species *P. thivervalensis* was reported to be isolated from the roots of *Brassica napus* and *Arabidopsis thaliana* [51], and is an important member of soil microbial communities [49]. *Pseudomonas* have also been implicated to be a plant growth-promoting rhizobacteria (PGPR) and have been associated with plant growth, control of pathogenicity [49] and aid in plant resilience under drought-stressed conditions [48, 50]. 

### Stress response genes identified in MAG-Pseudomonas enhanced drought tolerance

MAG-*Pseudomonas* has a total length of 6,777,975 bp, with 99 contigs and an N50 of 146,692 bp. The GC content of MAG-*Pseudomonas* is 61.1%. When annotated with the COG database, we noticed that it yielded 5,953 gene calls, and 4,924 were assigned at least one COG categorical function (Table 1).

**Table 1:** Bin assignment statistics to MAG-*Pseudomonas*: GC-content, N-50, number of contigs, percent completion, percent redundancy, and total length

	MAG-*Pseudomonas*
GC-content	61.11%
N-50	146,692 bp
Number of contigs	99
Percent Completion	100%
Percent redundancy	1.41%
Total length	6,777,975 bp

Previous studies [48, 50] based on 16S rRNA gene sequences have identified *Pseudomonas* in aiding the plant host to become more resilient under drought-stressed conditions. *Pseudomonas* is highly diverse in phylogeny and functions [45]. With the observation from the phylogenetic tree analysis that MAG-*Pseudomonas* belonged to the *Pseudomonas corrugata* phylogenomic subgroup, we ask what might be some potential functions of *Pseudomonas* that 1) enabled the survival of *Pseudomonas* under stressful conditions; 2) provided cues to how the *Pseudomonas* might assist in the stress tolerance of the associated plant host. We identified the universal stress protein (UspA) family (Table 2), which suggested that it had an important putative functional role in the survivability of MAG-*Pseudomonas* under a wide range of stress conditions [52, 53] which includes starvation of elements like nitrogen, carbon, phosphate, sulfate as well as heat exposures, etc. [53]. Drought might reduce growth yields [54], and UspA has been found to be abundant in growth-arrested cells [55]. We further identified aminocyclopropane-1-carboxylic acid (ACC) deaminase (*n* = 3) in the MAG-*Pseudomonas*, implying the putative gene could be essential in the modulation of the associated plant’s stress responses during drought conditions (Table 2, Supplementary Table S2). ACC deaminase is an enzyme known to enhance plant growth by cleaving plant-produced ACC, which decreases the ethylene production in the plant, thereby stimulating plant growth [18]. Moreover, the ACC deaminase has been extensively studied in *Pseudomonas* [56], and its role in stress tolerance is well documented [20, 21]. Putting it all together—1) MAG-*Pseudomonas* was closely related to *P. corrugata* in our phylogenomic analyses; 2) both *P. corrugata* [45] and MAG-*Pseudomonas* possess ACC deaminase; our results strongly suggested that MAG-*Pseudomonas* had functional potential in enhancing the associated plant’s resilience during drought stress.

**Table 2:**
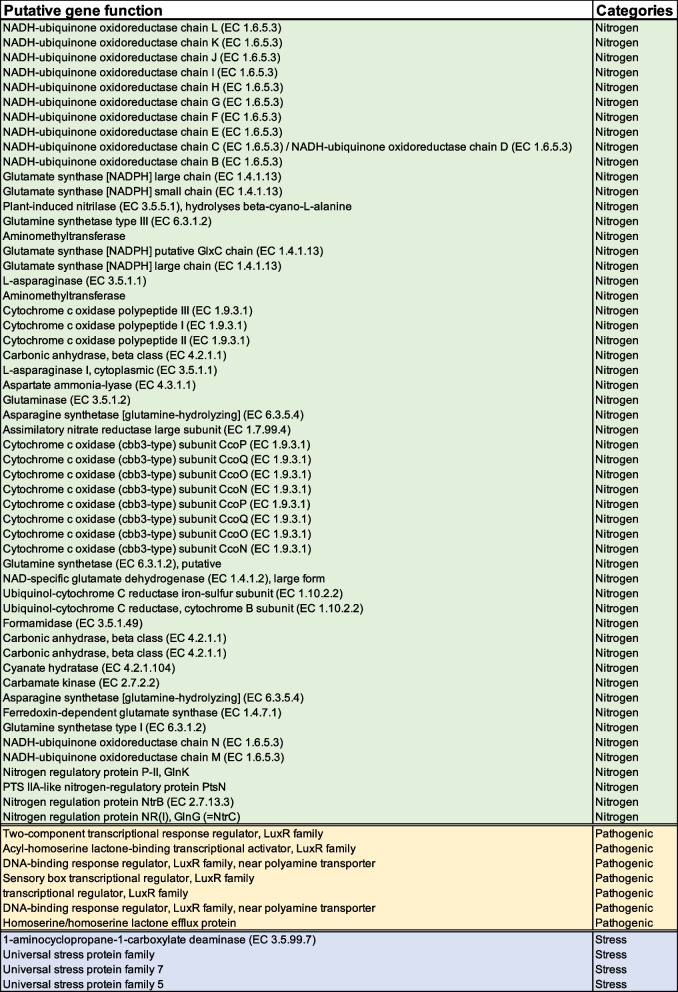
Annotated gene features in MAG-*Pseudomonas*. The gene features were annotated using the PATRIC portal. The table lists genes from stress, nitrogen transformation, and pathogenic behavior detected in MAG-*Pseudomonas*. This includes assimilatory nitrate reductase large subunit, ACC deaminase and LPQ island features

### Nitrogen transformation potential of MAG-Pseudomonas could enhance A. gerardii growth

Initial genomic analysis showed that our resolved MAG-*Pseudomonas* harbored several stress-response-related gene functions. Besides understanding microbial mechanisms of MAG-*Pseudomonas* resilience during drought-induced stress, we were also interested in gaining a deeper understanding of how the plant host could benefit from the *A. gerardii* and MAG-*Pseudomonas* interactions. We detected several putative gene functions that demonstrated the nitrogen transformation potential in our resolved MAG-*Pseudomonas*, which could contribute to the growth enhancement of the associated plant host, *A. gerardii.*

Nitrogen transformation genes—nitrogen regulation response regulator GlnG, nitrogen PTS system EIIA component, nitrogen regulatory protein PII, GlnK, and nitrogen regulation protein NtrB, that were detected in our resolved MAG-*Pseudomonas* (Table 2, Supplementary Table S2), has also been previously reported in other *Pseudomonas spp* [57–59]. All the nitrogen transformation gene functions that were detected in our MAG-*Pseudomonas* can be essential in helping to fulfill the plant host’s need for nitrogen, especially in N-depleted soils [60–63]. NtrB also plays a role in nitrogen metabolism and can regulate the nitrogen dynamics under nitrogen-deprived and enriched environments [64]. NtrC is another nitrogen metabolism regulator that contributes to nitrogen assimilation [58]. Similarly, nitrogen regulatory protein PII (GlnK) and nitrogen PTS system EIIA components are also involved in regulating nitrogen metabolism [65]. Assimilatory nitrate reductase catalytic subunit was also identified in this study which catalyzes the process from nitrate to nitrite [66] (Table 2).

**Fig. 2:**
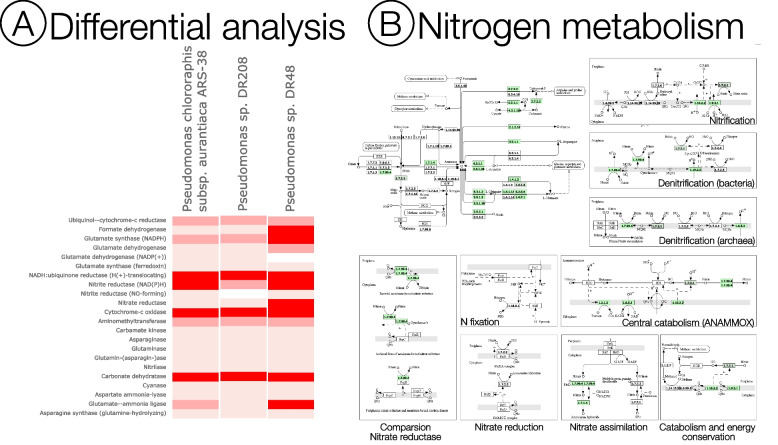
**A** Differential occurrence of the genes in MAG-*Pseudomonas* with *Pseudomonas chlororaphis* subsp. *aurantiaca strain* ARS 38, *Pseudomonas sp.* DR208 and *Pseudomonas sp.* DR48. The darker the highlight represents higher occurrences in the MAG-*Pseudomonas.* MAG-*Pseudomonas* showed a high occurrence of nitrate reductase and glutamate synthase in the MAG-*Pseudomonas* genome when compared with the other genomes. **B** Nitrogen metabolism pathways in MAG-*Pseudomonas* were detected based on a comparative pathway tool in PATRIC. MAG-*Pseudomonas* had 79 genes annotated to have enzyme classification numbers involved in the nitrogen metabolism pathway with 100% coverage. Nitrate reductase was found to be present in the principal nitrogen metabolism pathway, along with the denitrification pathway. Box numbers represent the Enzyme Commission number (E.C. number). The green colored boxes denote the annotated enzymes. The block comparison nitrate reductase corresponds to the dissimilatory nitrate reduction. The enzyme that the MAG-*Pseudomonas* has is the assimilatory nitrate reductase, so that could be identified in the nitrate assimilation block. The block nitrate assimilation corresponds to the assimilatory nitrate reductase. KEGG nitrogen metabolism pathways [72] were downloaded from PATRIC web portal. Copyright permission for use and modification were obtained from KEGG

We selected the 3 genomes from the Pathosystems Resource Integration Center (PATRIC) web portal. We used the comparative pathway tool in PATRIC, and identified 138 potential pathways of MAG-*Pseudomonas* based on genomic information from 3 *Pseudomonas* genomes—*Pseudomonas chlororaphis subsp. aurantiaca strain* ARS 38 isolated from the cotton rhizosphere, *Pseudomonas sp.* DR208 and *Pseudomonas sp.* DR48 isolated from the soybean rhizosphere. The criteria for the selection of these genomes were: 1) genomes were obtained from the rhizosphere; 2) genomes were complete and of high quality. The identified pathway classes included carbohydrate metabolism, lipid metabolism, metabolism of cofactors and vitamins, energy metabolism, nucleotide metabolism, biosynthesis of secondary metabolites, amino acid metabolism, xenobiotics biodegradation and metabolism, metabolism of other amino acids, glycan biosynthesis and metabolism, translation, signal transduction, and immune system (Supplementary Table S3). We further analyzed the differential occurrence of the genes in MAG-*Pseudomonas* and the 3 *Pseudomonas* genomes, and observed that there was a high occurrence of nitrate reductase and glutamate synthase in the MAG-*Pseudomonas* genome when compared with the other genomes (Fig. 2A). Nitrate reductase plays a key role in nitrogen transformation processes [66]. Given the importance of nitrogen-mediated microbial processes on plant growth [67], we analyzed the annotated MAG-*Pseudomonas* nitrogen transformation processes and showed that there were 79 annotated genes that were involved in the nitrogen metabolism pathway with 100% coverage. We identified several important genes in MAG-*Pseudomonas* associated with nitrogen transformation processes in the nitrogen metabolism pathway (Table 2, Fig. 2B). Glutamate synthases, identified in MAG-*Pseudomonas* are actively involved in ammonia assimilation pathways in bacteria [68] (Table 2), while glutamate dehydrogenase has a prominent role in nitrogen assimilation and is capable of maintaining the balance of carbon and nitrogen [69] (Table 2). We identified a wide range of genes involved in nitrogen transformation processes in MAG-*Psuedomonas* that could increase the nitrogen availability to the plant host [70, 71]. Putting it all together, our resolved MAG-*Pseudomonas* with its potential in microbial-driven nitrogen transformation processes could play a critical role in the regulation of primary productivity of its plant host, *A. gerardii,* even during times of drought-induced stress.

### MAG-Pseudomonas is essential to understand the resilience of the host plant under abiotic stress

**Fig. 3:**
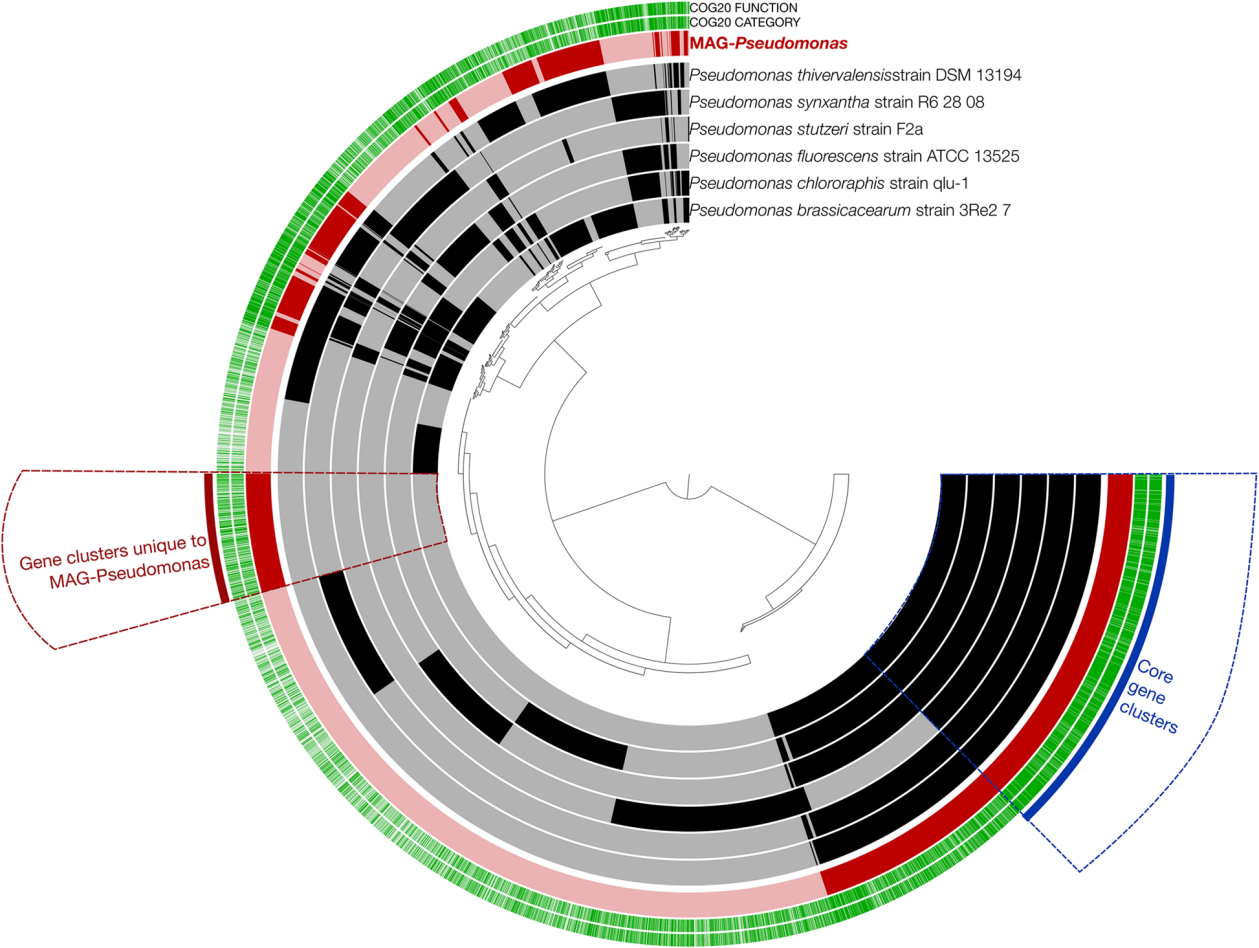
Pangenomic analysis of MAG-*Pseudomonas* with 6 related *Pseudomonas* genomes. Each layer represents a genome, and the region highlighted shows “gene clusters that was unique to MAG-*Pseudomonas”* and “core gene clusters” that was present in 100% of all the genomes. The highlight in the genomes represents the presence of the gene clusters. Tree in the middle represent the clustering of the gene clusters based on presence and absence of the genes. The two outer ring shows the presence or absence of annotation of COG function and category in the gene clusters. The genomes include *Pseudomonas thivervalensis* strain DSM 13,194 (Accession number: NZ_LT629691), *Pseudomonas synxantha* strain R6 28 08 (Accession number: NZ_CP027756), *Pseudomonas stutzeri* strain F2a (Accession number: NZ_AP024722), *Pseudomonas fluorescens* strain ATCC 13,525 (Accession Number: NZ_LT907842), *Pseudomonas chlororaphis* strain qlu-1 (Accession Number: NZ_CP061079), and P*seudomonas brassicacearum* strain 3Re27 (Accession Number: NZ_CP034725). All the genomes were downloaded on April 29, 2022

Our genomic analysis revealed several stress response and nitrogen transformation functional potential, but is there any niche specificity for MAG-*Pseudomonas*? We used a pangeomic analysis to compare the shared and unique gene functional potential of MAG-*Pseudomonas* and 6 related *Pseudomonas* genomes. We selected *Pseudomonas* genomes from RefSeq that have complete chromosomes [73], and had been subjected to rounds of quality check and consistent gene annotation [74], in order to ensure more conclusive pangenome analysis. Our analysis yielded 39,798 genes across the 7 genomes, with a total of 12,473 gene clusters. We used hierarchical clustering to group the gene clusters, showing similar distribution patterns across the 7 genomes (Fig. 3, Supplementary Table S4). Our pangenomic analyses identified a collection of 2,112 core gene clusters that occurred in 100% of all *Pseudomonas* genomes, and 719 gene clusters that only occurred in the MAG-*Pseudomonas* genome. The proportion of genes with functional annotation varies between the core and accessory clusters of MAG-*Pseudomonas.* We noticed that there were 94.4% of core gene clusters annotated with gene functions, using NCBI’s Clusters of Orthologous Groups (COGs) database, while only 63.6% of the gene clusters in the accessory clusters of MAG-*Pseudomonas* were annotated (Fig. 3, Supplementary Table S4).

We identified several stress response and nitrogen transformation genes in the core cluster in the pangenomic analysis which again reiterated our hypotheses that MAG-*Pseudomonas* might have microbial mechanisms that enhanced its survivability and would contribute to the plant hosts’ well-being under abiotic stress conditions (Fig. 3, Supplementary Table S4). The genes that were identified were predicted universal stress protein E, nucleotide-binding universal stress protein UspA family (UspA), desiccation stress tolerance protein with LEA/WHy domain (LEA), and universal stress protein A. (Fig. 3, Supplementary Table S4). Desiccation stress tolerance proteins with LEA/WHy domain (LEA) is suggested to confer a broad range of stress response function to bacteria such as *Escherichia coli* [75], while genes corresponding to a WHy protein homologue have been identified in both archaea and bacteria including *Pseudomonas* [76, 77] although the specific function in *Pseudomonas* is still incomprehensible. Our findings in MAG-*Pseudomonas* and 6 related genomes provided insights into potential gene functions in *Pseudomonas* that could be instrumental in providing resilience against drought induced stress. We also identified a set of universal stress proteins (UspA, UspE), which belonged to bacterial universal stress proteins, and were produced under stressful conditions [55]. We also identified other genes—YaaA (oxidative stress); TypA/BipA (general stress-response regulator, [78]; BolA (family transcriptional regulator, [79]; and Ribosomal protein L25 (general stress protein Ctc) (RplY) [80], that demonstrated the potentiality of *Pseudomonas* to elicit one or more microbial mechanisms to become more resilient when subjected to abiotic stresses. Similar to stress response genes, our findings, which identified numerous nitrogen transformation gene functions (Fig. 3, Supplementary Table S4), in the pangenome analysis corroborate with our MAG-*Pseudomonas* genome analysis that *Pseudomonas* might have the capability to contribute to the resilience and well-being of the plant host under environmental stresses.

Besides the core-clusters gene functions, we also observed genes related to chemotaxis in the MAG-*Pseudomonas* accessory gene-clusters. We detected genes corresponding to methyl-accepting chemotaxis protein and chemotaxis protein CheD (Supplementary Table S4). Our resolved MAG-*Pseudomonas* might show chemotaxis towards certain amino acids by using methyl-accepting chemotaxis proteins [81], as these bacterial cells are known to methylate the methyl-accepting chemotaxis proteins when adapting to environmental repellents and attractants [82]. Similarly, CheD chemotaxis proteins might be used by MAG-*Pseudomonas* to attract or evade various environmental stimuli [83–85]. Our MAG-*Pseudomonas* also had a gene corresponding to insecticidal toxin complex protein TccC. These proteins exhibit toxicity to a wide range of insects that could be utilized in designing strategies for crop protection [86]. Interestingly, we also identified the pathogenicity LPQ (lipopeptide/quorum-sensing) island that is also present in the closely related *Pseudomonas sp.* [45, 87–90]. This genetic island is characterized by the presence of luxR genes and acyl-homoserine lactone (AHL) efflux protein [45]. Both these features were identified in our MAG-*Pseudomonas* (Table 2, Supplementary Table S2)*.* The existence of such quorum-sensing machinery could be used by these specific groups of bacteria to respond to quorum sensing signals and demonstrate as markers of pathogenic behavior to plants or antifungal activity [87].

Tailoring SynComs is an important approach to provide insights into plant host-microbe interactions. Understanding the mechanisms and functions of host-associated microbial populations is particularly relevant in the construction of these plant-associated SynComs. Our study showed that MAG-*Pseudomonas* possessed putative genes that were involved in the function enhancing the resilience during drought-induced conditions, and might performed essential microbial functions for generating products related to the nitrogen cycle [91], which could be exploited by plant host and other host-associated microbes [92]. A SynCom consisting of six *Pseudomonas* strains isolated from the garlic rhizosphere has been reported to promote plant growth [93]. Thus, apart from the potential to contribute to the plant host’s well-being, our MAG-*Pseudomonas* might also be able to influence and interact with other bacteria [94], contributing to its role as an important member of the core rhizobiome along with other members such as *Streptomyces*, *Rhizobium*, *Burkholderia, Nitrosomonas*, *Nitrospira*, *Azospirillum*, *Bradyrhizobium,* and *Azotobacter *[95]. Overall, our study emphasized that the understanding of the MAG-*Pseudomonas* mechanism and functional potential might contribute to the successful construction of a SynCom that can benefit the plant-host during drought-induced stress [40].

## Conclusion

In this study, we used cultivation and metagenomic strategy to identify bacterial populations in the *A. gerardii* rhizobiome, and identified MAG-*Pseudomonas* as the candidate microbe that had significant functional potential in nitrogen transformation and stress response. In support of other studies, our study verified the abundance of MAG-*Pseudomonas* in the rhizobiome and suggested its potential pivotal role under drought conditions. In a continuing effort to understand the contributions of different microbiota in the plant rhizobiome, it is important to remember that identity and relative abundance alone may not truly reflect the relative functional importance of the bacterial population. Instead, understanding the functional role of the microbe during host-microbe and microbe-microbe interactions might provide more insights. The functional potential of our resolved MAG-*Pseudomonas,* resulting from a combination of conventional culturing and high-throughput analysis, showed the immense potential to inform and refine our efforts to dissect the mechanistic interaction taking place in the rhizobiome.

## Materials and methods

### Sampling, and cultivation of rhizosphere communities from soil samples

We collected *Andropogon gerardii* rhizosphere samples from a common garden in Colby at the Kansas State University Agricultural Research Center located in Thomas County (39°23′N, 101°04′W). Further information on the experimental layout, ecotypes, and sampling collections has been described previously [43]. In this comparative study, we selected rhizosphere samples from native dry (Hays, Kansas) and wet (Carbondale, Illinois) ecotypes growing in Colby for microbial cultivation. We separated bulk soil from the soil attached to the rhizosphere by handshaking the roots gently. We resuspended 0.1 g of the rhizosphere samples in 0.9 ml of Phosphate-Buffered Saline (PBS) [pH 7] buffer, serially diluted the samples (10^–1^—10^–6^), and spread 100 µl solution onto the Petri plates. We designed two culture conditions—R2A media (Teknova, USA) [96] and R2A media amended with a 36% Polyethylene Glycol 8000 (PEG) (Ψ = -1.54 MPa) to alter the media osmotic potential and to mimic absence and presence of water limitation, respectively [97, 98]. A similar range of PEG concentrations has been used to simulate dry environments in other studies [99, 100]. To prepare the R2A-PEG media, we dissolved 36% (w:v) PEG powder in autoclaved MilliQ water, allowing the mixed solution (20 mL) to sit on top of a pre-made R2A plate for 24 h to diffuse throughout the agar. After 24 h, we removed excess solution and spread 100 µl of the diluted soil culture on the surface of the agar. The prepared plates were incubated at 37℃ for 24–48 h until the appearance of the colonies. After the incubation period, we scraped all colonies by flooding the plate with 2 mL of sterile PBS buffer, transferred the liquid that contained microbes and stored at -20℃ until genomic DNA extraction. We were interested in capturing the bacterial communities that grew together in different conditions, so instead of picking individual colonies, we scraped all colonies from the individual plates to sequence the full genome(s) [44, 101]. Rhizosphere bacterial communities were cultivated from dry (R2A; *n* = 10 and R2A + PEG; *n* = 10) and wet ecotypic *A. gerardii* rhizosphere samples (R2A; *n* = 10 and R2A + PEG; *n* = 10).

### DNA extraction, shotgun sequencing, and analyses

We extracted the microbial DNA with the E.Z.N.A. Soil DNA Kit (Omega Bio-tek, Inc., Norcross, GA, USA) following the manufacturer’s protocol. Shotgun metagenomes were sequenced from the extracted samples on the Illumina NovaSeq 6000 (Illumina, San Diego, CA, United States), with a 150 bp paired-end sequencing strategy, with Nextera DNA Flex for library preparation and S1 flow cell. We used the program ‘iu-filer-quality-minoche’ [102] to process the short metagenomic reads and removed low-quality reads following criteria outlined in Minoche et al. ([103]. We organized the samples into 4 metagenomic groups (R2A + Wet ecotype; R2A + PEG + Wet ecotype; R2A + Dry ecotype; R2A + PEG + Dry ecotype) based on the cultivation conditions and ecotypes for co-assembling strategy. The quality-filtered short reads were co-assembled into longer contiguous sequences (contigs) using MetaHit v1.2.9 [104] with a minimum contig length of 1000 bp. We then used ‘anvi-gen-contigs-database’ in anvio ver 7.0 [105] to compute k-mer frequencies and identify open reading frames (ORFs) in the contigs using Prodigal v 2.6.3 [106], and recruited metagenomic short reads to the contigs. We then annotate the bacterial and archaeal single-copy genes using HMMER v3.2.1 [107]. NCBI’s Cluster of Orthologous Groups (COGs) [108] was used to assign functions to the ORFs. We mapped the metagenomic short reads to the contigs with Bowtie2 v2.3.5 [109], and converted mappings to BAM tiles with samtools v. 1.9 [110]. The converted BAM files were then profiled using ‘anvi-profile’ with a minimum contig length of 1,000 bp. We used CONCOCT v 1.1.0 [111] to bin the metagenomes, and used anvi’o ver 7.0 [105] to manually curate the bins into metagenome-assembled genomes (MAGs) that satisfied the conditions of > 70% completion and < 10% redundancy based on single copy genes. The MAGs were assigned to taxa using the single-copy core genes of bacteria and archaea. We further used ‘anvi-compute-genome-similarity’ to calculate average nucleotide identity (ANI) [112], using PyANI v0.2.9 [113] to compare the resolved MAGs and identify non-redundant MAGs based on 95% ANI [114]. We analyzed the resolved MAG occurence in a sample with the “detection” metric, ​​detailing how much of the contig recruited reads to it. We considered a MAG as detected in a metagenome if the detection was > 0.25, which is an appropriate cutoff to eliminate false-positive signals in read recruitment results.

### Phylogenetic, pathway, and pangenomic analyses

Among the resolved MAGs, there was a MAG of interest for this study: MAG-*Pseudomonas.* The selected non-redundant MAG was analyzed by the Similar Genome Finder service that uses the MinHash on the Pathosystems Resource Integration Center (PATRIC) web portal [115, 116]. Similar genomes deposited in public databases were obtained and used to estimate the genome distances to the MAG-*Pseudomonas*. We constructed a phylogenetic tree for the selected non-redundant MAG and 40 closely related genomes. The genome status of the 40 genomes was either complete or at the levels of whole genome sequencing. The workflow used the PATRIC Codon Tree Service which used the amino acid sequences from a well-defined database of global protein families [117]. In our workflow, we used amino acid sequence files for each genome. For tree construction, genomes were used with small set of potential outgroup genomes. It was a two-step process that we followed to identify the sets of homologous proteins. For the first step, a single genome from each distinct species was selected, and then aligned against each other using the BLAT alignment tool [118]. We clustered the top-scoring hits with Markov Cluster (MCL) Algorithm which defined the initial seed sets for determining the homologous groups [119]. In the second step, Multiple Sequence Comparison by Log-Expectation (MUSCLE) was used to align the seed sets [120]. Hidden Markov Model (HMMs) were built using the hmmbuild. All genomes including the outgroup pool were searched with hmmsearch. Homologous groups were then defined by hmmsearch top hits. Outgroup genomes were selected from the pool of outgroup candidates that were based on the total hmmsearch score. In the next stage, homologous groups were filtered for the taxon coverage, and MUSCLE was used to align the groups. Gblocks eliminated the poorly aligned regions, and the remaining well-aligned regions were concatenated into a long single alignment. The main phylogenetic tree was then estimated from the long single alignment using FastTree [121]. Bootstrap values can often be overly optimistic for long fragments. In our case, we used random samples of 50% of the homologous groups that were used for the main tree. This workflow was termed gene-wise jackknifing. In the final step, hundreds of the 50% gene-wise jackknife trees with the support values indicated the times a particular branch was observed in the support trees. We used the RAxML program v 8 [122] to construct a tree based on the pairwise differences between the aligned protein families of the selected sequences. For inferring the trees, the maximum likelihood method was used. We used the comparative pathway tool of PATRIC to predict the metabolic pathways in our selected MAG. The comparative pathway service of the PATRIC portal allowed us to identify the set of pathways that was based on EC number, taxonomy, pathway name, pathway ID and/or the annotation type. In the comparative analysis tool, we compared the MAG-*Pseudomanas* genome with the genome group that contained the *Pseudomonas* genomes isolated from rhizospheres of cotton and soybean using the select genome and select genome group options available under the comparative pathway service. The rationale behind using these *Pseudomonas* genomes was because these genomes were also isolated from the rhizosphere, giving us the opportunity to compare different *Pseudomonas* strains that were isolated from rhizospheres from different plant hosts. The criteria that we used for the genome selections were (1) genomes must be complete (2) genomes were of high quality (3) genomes isolated from the rhizosphere. That would allow us to have a comparative study between the *Pseudomonas* isolated from the rhizosphere of *A. gerardii* with *Pseudomonas chlororaphis subsp. aurantiaca* strain ARS 38 isolated from cotton rhizosphere and *Pseudomonas sp.* DR208 and *Pseudomonas sp.* DR48 from the soybean rhizosphere. We focused on the nitrogen metabolism pathway that we selected from the list of pathways available for MAG-*Pseudomonas* and the other *Pseudomonas* genomes. KEGG maps and heat maps of the nitrogen metabolism pathway were generated in the PATRIC portal.

We downloaded 6 related *Pseudomonas* genomes from NCBI RefSeq [74] and performed pangenomic analyses using anvi’o workflow [105, 123]. The criteria that we set up for efficient pangenomic analyses is to download only the genomes of *Pseudomonas* that were complete. The workflow uses BLASTP [124] to compute amino acid level similarities between all possible ORF pairs. We then used Markov Cluster Algorithm (MCL) [119] to group ORFs into homologous gene clusters and aligned amino acid sequences in each gene cluster using MUSCLE for visualization [120]. We determined the core gene clusters of the MAG-*Pseudomonas* and the 6 additional, available *Pseudomonas* genomes, as well as the accessory gene cluster of MAG-*Pseudomonas.*

## Supplementary Information

**Additional file 1:** **Supplementary Table S1.** Non-redundant MAGs and taxonomic identity.

**Additional file 2:** **Supplementary Table S2.** Gene functions of MAG-Pseudomonas.

**Additional file 3:** **Supplementary Table S3.** Pathways identified in MAG-Pseudomonas.

**Additional file 4:** **Supplementary Table S4.** Shared and unique gene clusters identified in pangenomic analysis of MAG-Pseudomonas with 6 related Pseudomonas genomes.

## Data Availability

The raw data used in this study are publicly available at NCBI under the project accession PRJNA844897. Analyzed data in the form of databases and fasta files can be found at figshare https://doi.org/10.6084/m9.figshare.20005550.

## Abbreviations

USA: United States of America
PGPB: Plant Growth-Promoting Bacteria
SynComs: Synthetic Communities
MAG: Metagenome-Assembled Genome
R2A: Reasoner's 2A Agar
PEG: Polyethylene Glycol
ANI: Average Nucleotide Identity
PGPR: Plant Growth-Promoting Rhizobacteria
COGs: Cluster of Orthologous Genes
rRNA: Ribosomal Ribonucleic Acid
USP: Universal stress protein
PBS: Phosphate-Buffered Saline
ORFs: Open Reading Frames
PATRIC: Pathosystems Resource Integration Center
DNA: Deoxyribonucleic acid
anvi’o: An analysis and visualization platform for 'omics data
RAxML: Randomized Axelerated Maximum Likelihood
NCBI: National Center for Biotechnology Information
KEGG: Kyoto Encyclopedia of Genes and Genomes
RefSeq: Reference Sequence
MCL: Markov Cluster Algorithm
